# Voluntary or Forced: Different Effects of Personal and Social Norms on Urban Residents’ Environmental Protection Behavior

**DOI:** 10.3390/ijerph17103525

**Published:** 2020-05-18

**Authors:** Guanglin Bai, Yun Bai

**Affiliations:** Management School, Jiangsu University, Zhenjiang 212013, China; by9423@yeah.net

**Keywords:** personal norms, social norms, environmental protection behavior, difference analysis

## Abstract

It is well known that environmental protection behaviors are influenced by both individual internal motivation and external environmental pressure, but few studies have looked at the two kinds of factors together. In order to study the influence mechanism of these two kinds of factors on the environmental protection behavior of urban residents, especially the difference between these two kinds of factors, we take personal norms and social norms as independent variables into the theoretical model. Results based on survey data of 731 urban residents revealed that personal norms and social norms both are positively associated with environmental protection behavior. Moreover, environmental protection willingness was found to mediate the relationship of personal and social norms with environmental protection behavior. We also found that the direct and indirect influences of personal norms on environmental protection behavior are greater than that of social norms. Further, the study revealed that cost consciousness moderates the relationship between personal norms, environmental protection willingness, and environmental protection behavior. Our results suggest that personal norms have a greater impact on environmental protection behavior than social norms. Therefore, we need to make greater efforts to promote environmental education and cultivate young people’s sense of environmental responsibility from an early age. At the same time, it is necessary to maintain appropriate environmental pressure and reduce the environmental cost in the daily life of residents.

## 1. Introduction

Environmental problems have been the constant focus of global attention in recent years. In March 2019, at the Fourth United Nations Environment Assembly, the United Nations Environment Program released the sixth edition of the Global Environment Outlook (GEO6). The report states that the damage to the planet is so severe that, unless urgent and stronger action is taken to protect the environment, the planet’s ecosystems and the course of sustainable human development will be increasingly threatened. With the growth of economy and people’s income, our attitude towards environmental problems should not be indifferent. It is necessary to take some actions to protect our environment [1]. However, even though the concept has been continuously advocated and promoted at a global level, many people still practice “free riding” behavior in their daily lives and turn a blind eye to the environmental problems around them. Encouraging every citizen to take positive actions and practice environmental protection is an important link to balancing the ecology and reducing pollution. However, while environmental protection is a key issue, promoting environmental improvement is quite difficult. Therefore, how to promote the implementation of environmental protection awareness in urban residents and encouraging them to practice environmental protection is a new problem in this new era of economic and social development.

Nowadays, many scholars have noticed and studied the antecedents of environmental protection behavior and have achieved some notable results. Factors such as psychological consciousness [2], social responsibility [3,4], personal values [4,5], self-direction [4], knowledge and attitude [6], social norms [7], and even religion [8] and national culture [9], have all been proved to have a significant influence on people’s environmental protection behavior. These influencing variables fall into two main categories: individual internal factors and external environmental factors. Obviously, environmental protection behavior is the result of a combination of these two types of factors. However, previous research has focused more on a certain variable or class of variables to explore their impact on environmental behavior. For example, Wang found that recycling behavior of city residents was determined by five factors: perception, knowledge, responsibility consciousness, attitude, and age [2], all of which are individual internal variables. Analogously, Su et al. found social responsibility contributed to resident environmentally responsible behavior [3] and the results of Andreas et al. showed that personal values had a positive impact on environmental behavior [4]. In addition, there are many studies on the impact of external environmental factors on environmental behavior. Huber et al. explored the relationship between social norms and pro-environmental behavior [7], and Zheng et al. found that social interaction had a promoting effect on public environmental protection behavior [10]. These studies have revealed the determinants of environmental behavior to some extent, but little attention has been paid to the interaction between influencing factors, especially the comparative study of the effects between different internal and external influencing factors.

Previous literature has provided evidence that the moral value of the individual has a significant impact on environmental behavior [11,12]. The Norm Activation Model holds that personal norms are an individual’s sense of self-ethical obligation to perform a behavior [13]. Previous research efforts also generally agree that personal norms directly affect environmental intention and behavior [14,15]. Meanwhile, many studies have noted that individual behavior is also affected by group behavior [10,16]. When under pressure from the people who surround them, individuals are more easily affected, and they will make corresponding changes. In the theory of planned behavior, subjective norms are defined as the social pressures felt by individuals [17]. This can be seen as social norms, which also affect intentions and behaviors [18,19]. In addition, cost is usually an important factor that affects behavior; cost may affect the degree of the impact of other factors [20]. However, previous literature lacks a combination of the factors of responsibility and the influence of surrounding people, and the differences between them. Cost conscious considerations should also be included in this framework.

Based on the above, this study selects personal norms and social norms as the measurement index of internal and external factors. In addition, cost consciousness is used as a moderator to environmental protection behavior. First, we aimed to explore the influence mechanism of personal norms and social norms on environmental protection behaviors. Second, we tried to compare the impact of personal norms and social norms on environmental behavior. Third, we investigated moderating the effect of cost consciousness on the relationship between personal and social norms and environmental protection behavior.

Our main contribution is to examine the positive impact of personal norms and social norms on environmental protection behaviors. On this basis, we have established an impact mechanism model and focused on comparing the impact of personal and social norms on environmental protection behaviors. We attempt to find a process mechanism to promote residents’ environmental protection behavior from “voluntary” or “forced” approaches, so as to find more effective measures for continuous improvement of environmental protection behavior.

The rest of this paper is organized as follows. In Section 2, we proposed hypotheses and constructed a theoretical framework. In Section 3, we illustrated the methodology including measurement of variables, sample and data collection. In Section 4, we reported the results of the analyses. The last section drew conclusions and discussion.

## 2. Hypothesis and Theory Framework

### 2.1. Personal Norms, Social Norms, and Environmental Protection Behavior

Behaviors are influenced by internal and external factors. These two factors are different and complementary to each other, and they jointly promote the occurrence and development of different things. Environmental protection is a socially beneficial behavior. However, for individuals, by activating their biosphere value or by enhancing environmental self-identity, many actions that are beneficial to the environment can be stimulated [21]. Therefore, from this perspective, environmental protection behavior is more affected by personal internal factors. According to the Norm Activation Model, personal norms refer to the individual’s sense of self-ethical obligation to perform an action. As such, personal norms are a kind of self-expectation, and they reflect the individual’s sense of responsibility for implementing specific actions [13]. Personal norms are stronger predictors of environmental behavior than other psychological variables (e.g., personal values, environmental concerns) or social-demographic characteristics [22]. Therefore, we use personal norms as a measure of internal environmental responsibility.

Personal norms have a positive impact on individual behaviors. This has been confirmed by many scholars. For example, Tina’s research has proved that personal norms are the main determinants of individual clothing consumption intention and behavior [23]. Doran has found that personal norms have the strongest correlation with eco-friendly travel choices [22]. Therefore, we believe that the higher the personal norms of residents are, the better their environmental protection behavior will be. We thereby propose Hypothesis 1.

**Hypothesis 1** **(H1).** *Personal norms have a positive impact on environmental protection behavior*.

From the perspective of external factors, although environmental protection behavior is not mandatory, it is bound to be affected by the environmental behavior of the people around any given individual and the corresponding social atmosphere. According to the theory of planned behavior, subjective norms refer to the social pressures that individuals feel with regard to engaging or not engaging in something, including the behavior and expectations of other individuals or organizations [17]. These factors influence an individual’s decision making and whether they choose to take action [24,25]. Social norms are ethical standards and codes of conduct commonly accepted by society; they can be seen as a kind of social pressure. People usually take into account the gap between their behavior and others and social norms before engaging in any behavior. Especially in society, public pro-environmental behavior has characteristics of externality and interactivity. As such, the behavior of others could affect the pro-environmental behavior of individuals [10]. Individuals are also more willing to produce behaviors that are consistent with the groups they are in, for the purpose of social support and to fulfill social needs [26]. For example, social norms have a positive impact on promoting physical exercise [27]. Huber et al. found that social descriptive norms have a strong role in clarifying to citizens what their compatriots are doing and can promote community recycling behavior [7]. Therefore, the views and behaviors of surrounding people and the public with regard to environmental protection will have a non-negligible impact on every resident living within that social sphere. Individuals will adjust their behaviors according to the positive or negative responses of the people around them, and even the public. In other words, the stronger the social norms felt by urban residents are, the easier it will be for environmental protection behavior to occur. Therefore, we propose Hypothesis 2.

**Hypothesis 2** **(H2).** *Social norms have a positive impact on environmental behavior*.

### 2.2. Environmental Protection Willingness and Environmental Protection Behavior

Willingness is the psychological tendency and motivation of an individual to perform a certain behavior. Willingness also reflects the tendency of a person to give time and effort to accomplish something. The theory of planned behavior believes that willingness directly affects behavior. The stronger the individual’s willingness to do something is, the higher the possibility of implementation will be. Wang et al. found that individuals’ willingness to turn to green traffic positively influences green traffic behavior [28]. By the same token, the stronger the residents’ willingness to protect the environment is, the more likely it is that environmental protection behaviors will occur. Moreover, this conclusion has also been confirmed by many studies. For example, Cai et al. concluded that the low-carbon commuting behavior of bicycle sharing in China is directly affected by the cyclists’ low-carbon willingness [29]. Shen et al. found that low-carbon behavioral intention can directly promote the low-carbon production behavior of rice farmers [30]. From this, we propose Hypothesis 3.

**Hypothesis 3** **(H3).** *Environmental protection willingness has a positive impact on environmental protection behavior*.

The theory of planned behavior proposes that attitudes, subjective norms, and perceived behavioral control collectively affect behavioral intentions and then promote behaviors through behavioral intentions [17]. It can be seen that behavioral intention is the most direct factor affecting behavior, while other variables will affect behavior through their mediating effect. Oteng-Peprah et al. found that personal norms and social pressures have a positive and significant impact on householders’ intentions to adopt waste-water treatment and reuse systems [31]. Cowan and Kinley showed that the social pressure to act in an environmentally friendly manner can influence the intention to purchase environmentally friendly clothing and then predict future environmentally friendly purchase behaviors [32]. Through a questionnaire survey, He et al. found that environmental knowledge, perception of environmental problems, and awareness of environmental responsibility all influence environmental protection behaviors through the mediating role of environmental protection willingness [33]. Therefore, an individual’s sense of environmental responsibility and external environmental pressure must first act on their environmental protection willingness, which can then be translated into environmental protection behavior. Hence, we propose Hypotheses 4a and 4b.

**Hypothesis 4a** **(H4a).** *Environmental protection willingness mediates the relationship between personal norms and environmental protection behavior*.

**Hypothesis 4b** **(H4b).** *Environmental protection willingness mediates the relationship between social norms and environmental protection behavior*.

### 2.3. Comparison of the Effect of Personal Norms and Social Norms on Environmental Protection Willingness and Behavior

Materialist dialectics hold that the development of things is caused by internal and external causes, but their roles and status are different. The internal cause is the main driving force behind the development of things. The external cause generally needs to function through the internal cause. The internal cause is the first cause and the external cause is the second cause. Kallgren et al. also argued that internal rather than external processes regulate behavior [34]. In terms of the impact on residents’ environmental protection willingness and behavior, personal norms reflect the factors associated with internal responsibility, and social norms reflect the factors associated with external social pressure. The former may have a greater impact than the latter. There are studies showing that social norms seem to affect behavior not only directly, but also through personal norms [14,35]. Doran and Larsen found that, compared with social norms, personal norms explain a relatively large part of variance in the intention to choose eco-friendly tourism [22]. Janmaimool found that personal norms have a direct effect on waste management behaviors, whereas social norms did not directly influence waste management behaviors [36]. Personal norms also have a greater impact on people’s willingness to buy local food than social norms [37]. On the other hand, the forced behavior of residents engaged in environmentally friendly activities under pressure is usually not as good or positive as would be the case with voluntary behavior. Even if the pressure is not properly applied, unfriendly environmental thinking and the corresponding negative behavior will occur. Therefore, compared with social norms, personal norms have a greater impact on environmental protection willingness and behavior. Hence, we propose the Hypotheses 5a–5c.

**Hypothesis 5a** **(H5a).** *The influence of personal norms on environmental protection willingness is greater than the influence of social norms*.

**Hypothesis 5b** **(H5b).** *The influence of personal norms on environmental protection behavior is greater than the influence of social norms*.

**Hypothesis 5c** **(H5c).** *The influence of personal norms on environmental protection behavior through environmental protection willingness is greater than the influence of social norms*.

### 2.4. The Moderating Effect of Cost Consciousness

For a rational-economic man, cost paid is an important consideration for implementing specific behaviors. Especially in China, the income level of urban residents is not very high, and economic factors such as price and cost are important factors to consider before engaging in an activity. When engaging in environmental protection can reduce energy consumption and bring about corresponding cost savings, people will tend to engage in environmental protection. For example, Roos and Tjarnemo found that high prices are one of the main reasons that hinder consumers from choosing products with carbon labels [38]. Zhang et al. also found that consumers’ perceived prices significantly and positively affect their purchasing attitudes towards energy-saving appliances [39]. In the study of Tobler et al., the perceptions of cost and benefit proved to be the strongest predictor of support for climate policy measures [40]. Li et al. proved that the price of low-carbon products and economic incentive measures are important factors that influence consumers’ willingness to buy low-carbon products [41]. Environmental protection behaviors include low-carbon behaviors and conservation behaviors, which can reduce people’s economic losses and save daily expenses to a certain extent. Cost awareness refers to awareness of the existence and importance of costs and the degree of care for cost savings [42,43]. Therefore, this study takes cost consciousness as a moderating variable to explore the complex relationship between personal norms, social norms, environmental protection willingness, and environmental protection behavior.

Cost consciousness moderates the impact of environmental protection willingness on environmental protection behavior. When consumers found that environmental protection behaviors (such as the use of energy-saving appliances), can reduce unnecessary consumption and the associated costs, the possibility of urban residents turning environmental protection willingness into environmental protection behaviors increases. In the same way, if people don’t care about whether engaging in environmental protection behavior can reduce costs, (even if they have a desire to do so), it is not clear that their desire will actually translate into environmental protection behavior. Therefore, when cost consciousness is low, environmental protection willingness will have less of an impact on environmental protection behavior. This has also been confirmed by many scholars. For example, Wang et al. found that individual implementation cost is a moderating variable that affects the relationship between low-carbon psychological awareness and low-carbon consumption patterns [44]. Therefore, this study believes that the higher the cost consciousness of environmental protection behavior is, the more obvious the positive effect of environmental protection willingness on environmental protection behavior will be. Hence, we propose Hypotheses 6a–6c.

**Hypothesis 6a** **(H6a).** *Cost consciousness moderates the relationship between personal norms and environmental protection behavior. When cost consciousness is high, the positive relationship between personal norms and environmental protection behavior is stronger, and vice versa*.

**Hypothesis 6b** **(H6b).** *Cost consciousness moderates the relationship between social norms and environmental protection behavior. When cost consciousness is high, the positive relationship between social norms and environmental protection behavior is stronger, and vice versa*.

**Hypothesis 6c** **(H6c).** *Cost consciousness moderates the relationship between environmental protection willingness and environmental protection behavior. When cost consciousness is high, the positive relationship between environmental protection willingness and environmental protection behavior is stronger, and vice versa*.

**Figure 1:**
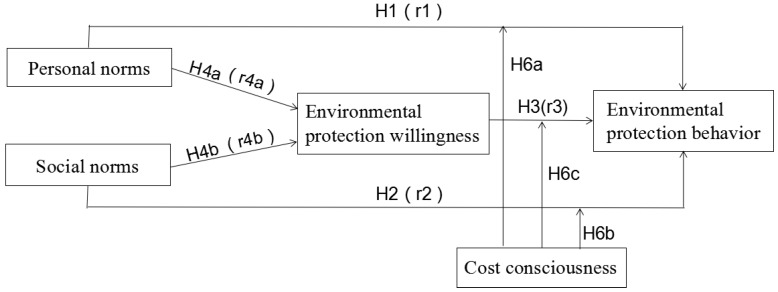
Theoretical model.

Based on the above research hypotheses, this study builds a model of the factors that influence urban residents’ environmental protection behavior (Figure 1).

## 3. Methodology

### 3.1. Measurement of Variables

In order to ensure the reliability of the questionnaire design, we read a large number of relevant studies before designing the questionnaire, and carefully discussed and studied each topic. All the items were modified to fit the problems in this study on the basis of the existing mature scales.

The measurement of environmental protection behavior draws on the research of Hong Kong scholar Chan [45], using eight items to measure the degree of environmental protection behavior, e.g., “actively participating in complaints that require environmental issues to be resolved”.

The measurement of personal norms draws on the research of Abrahamse and Steg [46], with a total of five items, e.g., “I have the responsibility to do my best to save resources and protect the environment.”

The measurement of social norm refers to the Value-Belief-Norm theory of Stern [47] and the research of Sun [48], and Zheng et al. [49], combined with concepts to carry out self-design. Finally, three items are established, e.g., “Almost everyone around us thinks that environmental protection, low-carbon, and energy-saving measures should be taken in life”.

The measurement of environmental protection willingness draws on the research of Chan [45]. Four items are used for measuring, e.g., “I would like to participate in environmental protection, low-carbon and energy-saving activities”.

The measurement of cost consciousness refers to the scale of Stern [47], which was revised according to the actual situation of Chinese residents. Finally, four items are established, e.g., “I take environmental protection and energy-saving actions to save money”.

Environmental protection behavior was measured using Likert 3-point scales, where 1 means “never”, 2 means “occasional”, and 3 means “often”. The other four variables used a Likert 5-point scale, with 1 to 5 representing “strongly disagree”, “disagree more than agree”, “unclear”, “agree more than disagree”, and “strongly agree”.

### 3.2. Sample and Data Collection

We conducted a random survey of Zhenjiang city residents in supermarkets, fast food restaurants, bus stations, cinema, library and other places. Zhenjiang is a typical city in eastern China, and the survey site guarantees the randomness of the sample. A total of 1000 questionnaires were distributed from August to September 2018, and 731 valid questionnaires were recovered, for an effective rate of 73.1%.

In order to improve the quality of the survey, the questionnaire was conducted anonymously “one-on-one”. Firstly, the questionnaire sender introduced the purpose and significance of the survey to the respondents, and after obtaining the consent of the respondents, the questionnaire was issued for them to fill in. At the same time, in order to facilitate the respondents to fill in the questionnaire and obtain the cooperation of the respondents, each respondent presented a neutral pen or other small gifts worth 10 yuan.

The demographic characteristics of the survey were as follows. The participants included 60.1% men and 39.9% women. Further, with regard to age, 3.6% were under 18, 51.4% were aged between 18 and 30, 30.4% were aged between 31 and 40, 12.3% were aged between 41 and 50, 2.3% were aged above 50. In terms of occupation, 3.7% were government staff, 40.8% were general worker or service worker, 7.9% were business executives, 4.7% were engineer, 5.3% were educator, scientist or environmental workers, 7.1% were private enterprise owners, 2.5% were retiree and the unemployed, 28% were others. Regarding monthly income, 9.3% were less than ¥1000, 32% were ¥1000–¥3000, 41% were ¥3000–¥5000, 12.9% were ¥5000–¥7000, 4.0% were ¥7000–¥10,000, 2.7% were over ¥10,000.

## 4. Results of Analysis

### 4.1. Reliability and Validity Analysis

Before the data analysis, we tested the reliability and validity of the scale and the measurement model. This was done to ensure the adequacy and appropriateness of the measurement scale used in this study in describing specific connotation concepts.

In terms of reliability, the Cronbach’s α of each scale was tested using SPSS. The results show that most of the Cronbach’s α of each scale are greater than 0.700, and the cost consciousness scale is 0.681, which is very close to 0.700. This result indicates that the scale used in this study has high reliability (Table 1).

In terms of validity, first, the overall fit of the model was tested with AMOS software, where χ^2^ = 742.542, df = 179, χ^2^/df = 4.148 < 5, RMSEA = 0.066 < 0.08, and CFI = 0.914 > 0.9, which shows that the measurement model used in this study has a good fit.

Secondly, after calculation (Table 1), the normalized factor load of each item is above 0.5, the combination reliability (CR) of each variable is greater than 0.8, and the average variance extraction amount (AVE) is above 0.5. These findings indicate that the potential variables of the scale have good convergent validity. At the same time, the square root value of the AVE of all latent variables is greater than the correlation coefficient with other latent variables. This indicates that the latent variables selected in this study have good discriminant validity (Table 2).

Furthermore, confirmatory factor analyses were conducted to test the discriminant validity of the study variables. We constructed nine alternative models to compare with theoretical models. M1, M2, and M3 were four-factor models, which combined personal norms and social norms, personal norms, and environmental protection willingness, and personal norms and cost consciousness, respectively. M4, M5 and M6 were three-factor models. In M4, personal norms, social norms and environmental protection willingness were combined into one factor. In M5, personal norms, social norms and cost consciousness were combined into one factor. In M6, personal norms, social norms and environmental protection behavior were combined into one factor. M7, M8 and M9 are two-factor models. In M7, personal norms, social norms, environmental protection willingness, and cost consciousness were combined into one factor. In M8, personal norms, social norms, environmental protection willingness and environmental protection behavior were combined into one factor. In M9, personal norms, social norms, cost consciousness and environmental protection behavior were combined into one factor. The results in Table 3 show that the five-factor model has the best model fit compared to other models (χ^2^ = 742.542, df = 179, χ^2^/df = 4.148 < 5, RMSEA = 0.066 < 0.08, and CFI = 0.914 > 0.9).

### 4.2. Hypothesis Testing

#### 4.2.1. Influencing Factors of Environmental Protection Behavior

The multiple linear regression method was used to explore the influencing factors of residents’ environmental protection behavior. Specifically, environmental protection behavior was taken as the dependent variable: personal norms, social norms, and environmental protection willingness were taken as independent variables. At the same time, gender, age, occupation, and monthly income were used as control variables in the regression equation.

In the regression model, we tested the VIF value of each variable in each model and marked the maximum VIF value in Table 4. The results show that each VIF value is less than 5, indicating that there is no collinearity between variables. The regression results are shown in Table 4. After controlling for gender, age, occupation, and monthly income, the impact of personal norms, social norms, and environmental protection willingness on environmental protection behavior reached a very significant level, with positive coefficients of 0.179, 0.147, and 0.237, respectively. Therefore Hypothesis 1, Hypothesis 2, and Hypothesis 3 are supported.

#### 4.2.2. Mediating Effect of Environmental Protection Willingness

The bootstrapping method was used to test the mediation effect. Bootstrapping takes the research sample as the sampling population. The return sampling method is used to repeatedly extract a certain number of samples from the research sample for estimation. The bootstrapping method avoids the problem in which the coefficient product test violates the distribution assumption. At the same time, this method avoids the problem of inconsistent results generated by different standard error formulas. Compared with other methods, the bootstrapping method has higher statistical power and is an ideal method for testing mediation effects.

We performed 1000 bootstrap re-sampling analyses on the data. The test results are shown in Table 5.

As can be seen from Table 5, the 95% confidence interval of the indirect effect of “personal norms → environmental protection willingness → environmental protection behavior” excludes zero. This finding means that environmental protection willingness plays a significant mediating role between personal norms and environmental protection behavior. Similarly, environmental protection willingness also plays a significant role in mediating between social norms and environmental protection behavior.

#### 4.2.3. Comparison of the Impact of Social and Personal Norms on Environmental Willingness and Behavior

The bootstrapping method was used to explore the effect of personal norms and social norms on environmental protection behavior and environmental protection willingness. The results are shown in Table 6.

As shown in Table 6, r1 and r2 represent the path coefficients of personal norms and social norms to environmental protection behavior; r3 represents the path coefficients of environmental willingness to environmental behavior, and r4a and r4b represent the path coefficients of personal norms and social norms to environmental protection willingness (Figure 1).

As can be seen from Table 6, when comparing the impact of personal norms and social norms on environmental protection behavior, the path coefficients between the two are positive and significant (r1-r2 = 1.308, *p* < 0.01). It can be seen that the impact of personal norms on environmental protection behavior is significantly greater than social norms. Therefore, Hypothesis 5b is supported. Then, comparing the impact of personal norms and social norms on environmental protection willingness, it is found that the difference between the path coefficients of the two is positive and very significant (r4a-r4b = 1.568, *p* < 0.01). This shows that the impact of personal norms on environmental protection willingness is significantly greater than social norms. Therefore, Hypothesis 5a is supported. Finally, comparing the indirect effects of personal norms and social norms on environmental protection behavior through the mediating effect of environmental protection willingness, it is found that the difference between the path coefficients of the two is positive and significant (r4a × r3-r4b × r3 = 1.450, *p* < 0.01). It can be seen, then, that the impact of personal norms on environmental protection behavior through environmental protection willingness is greater than the impact of social norms. Therefore, Hypothesis 5c is supported.

#### 4.2.4. Moderating Effect of Cost Awareness

Hierarchical regression equations are often used to test the moderating effects of variables. According to the moderating effect examination steps [50], we tested the role of environmental protection willingness. Model 1 only contains control variables such as gender and age. In Model 2, the independent variable of environmental protection willingness has been added. In Model 3, the moderator variable of cost consciousness is included. Finally, we centralized the environmental protection willingness and cost consciousness, and then constructed a product term of the two and put that product term into Model 4 for regression analysis. The regression results are shown in Table 7.

In the regression model, we tested the VIF value of each variable in each model and marked the maximum VIF value in Table 7. The results show that the maximum value of each VIF is less than 5, showing that there is no collinearity between variables. It can be seen from the results that the interaction between personal norms and cost consciousness is significant (β = 0.095, *p* < 0.05). There is also a significant interaction between environmental protection willingness and cost consciousness (β = 0.118, *p* < 0.01). However, the interaction between social norms and cost consciousness is not significant. This finding shows that cost consciousness has a significant moderating effect on personal norms and environmental protection behavior, as well as environmental protection willingness and environmental protection behavior. Moreover, the stronger the cost consciousness is, the greater the impact of personal norms on environmental protection behavior will be, and the greater the impact of environmental protection willingness on environmental protection behavior. However, in this study, cost consciousness had no moderating effect on the relationship between social norms and environmental protection behavior. This result may be due to the fact that under the special social and cultural background of China, urban residents are more concerned about the society and others’ opinions on them. Especially when everyone around them is doing something, people will still do whatever it takes to stay in line. Therefore, Hypotheses 6a and 6c are supported, and Hypothesis 6b is not.

**Figure 2:**
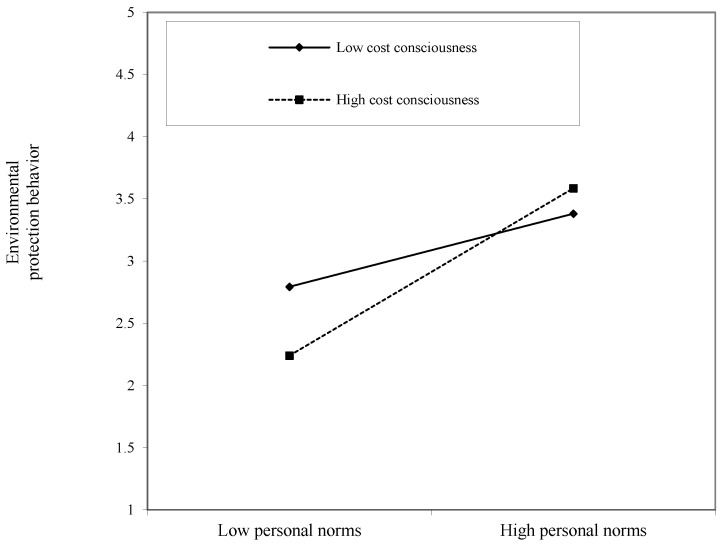
The moderating role of cost consciousness in the relationship between personal norms and environmental protection behavior.

**Figure 3:**
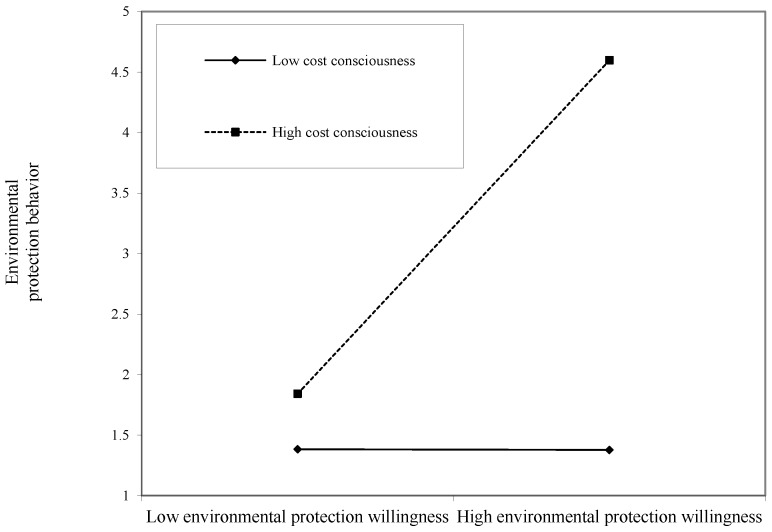
The moderating role of cost consciousness in the relationship between environmental protection willingness and environmental protection behavior.

In order to describe the moderating effect of cost consciousness more clearly, according to Cohen’s method [51], one standard deviation above and below the mean is used as the benchmark. The differences between the personal norms, environmental protection willingness, and environmental protection behavior at different levels of cost awareness are depicted as shown in Figure 2 and Figure 3.

As can be seen from Figure 2, regardless of the level of cost consciousness, the regression line slope of the relationship between personal norms and environmental protection behavior is positive; the slope of the straight line is higher when there is high cost consciousness. Therefore, cost consciousness positively moderates the relationship between personal norms and environmental protection behavior. 

As can be seen in Figure 3, when the level of cost willingness is high, the slope of the regression line of the relationship between environmental protection willingness and environmental protection behavior is positive. When the level of cost willingness is low, there is a weak negative relationship between environmental protection willingness and environmental protection behavior. It can be seen that the higher the cost consciousness is, the higher the regression line slope of the relationship between environmental protection willingness and environmental protection behavior. Therefore, cost consciousness positively moderates the relationship between environmental protection willingness and environmental protection behavior. This conclusion further supports the validity of Hypotheses 6a and 6c.

## 5. Discussion

First, personal norms, social norms, and environmental protection willingness all affect environmental protection behavior, which is consistent with previous research [7,22,23,27,28,29,30]. However, environmental protection willingness is the most important and direct influencing factor. Environmental protection willingness acts as a full mediator for the relationships between personal norms, social norms and environmental protection behavior. This conclusion also reaffirms the point that “the willingness of behavior to mediate between psychological variables and environmental behavior has become a consensus in the field of environmental protection research”, as proposed by Chen et al. [52]. Therefore, in order to promote environmental protection behavior, enhancing residents’ environmental protection willingness is a very effective link. 

Second, the impact of personal norms on environmental protection willingness and environmental behavior is greater than social norms. In other words, compared with the external environmental protection pressure, the urban residents’ sense of individual responsibility and values has a greater effect on their environmental protection behavior. Therefore, in order to enhance environmental protection willingness and encourage people to engage in more environmental protection actions, it is more effective to attempt to increase the level of residents’ internal values and sense of responsibility.

Third, cost consciousness positively moderates the relationship between personal norms and environmental protection behavior, environmental protection willingness and environmental protection behavior. Compared with low cost consciousness, when residents’ cost consciousness is higher, personal norms have a more positive impact on environmental protection behavior. On the contrary, if the level of cost consciousness is low, the impact will be weakened, or even reversed. However, the moderating effect of cost consciousness on the relationship between social norms and environmental behavior has not been confirmed, which shows that the impact of social norms on environmental protection behavior is not affected by cost awareness. Chinese people are high in collectivism, which is more about how others view their own behavior. This may be why the relationship between social norms and environmental behavior is unknot affected by other factors.

## 6. Conclusions

Based on survey data of 731 urban residents our study revealed that personal norms and social norms both were positively associated with environmental protection behavior. Moreover, environmental protection willingness was found to mediate the relationship between personal norms, social norms and environmental protection behavior. We also found that the direct and indirect influences of personal norms on environmental protection behavior were greater than these of social norms. Further, the study revealed that cost consciousness positively moderated the relationship between personal norms, environmental protection willingness and environmental protection behavior. Our results suggest that personal norms have a greater impact on environmental protection behavior than social norms. Therefore, we need to make greater efforts to promote environmental education and cultivate young people’s sense of environmental responsibility from an early age. At the same time, it is necessary to maintain appropriate environmental pressure and reduce the environmental cost in the daily life of residents.

## 7. Research Contributions and Limitations

### 7.1. Theoretical Contribution

This research contributes to the literature and practice in several ways. First, we unified internal responsibility factors and external environmental pressure factors into an impact mechanism model of environmental protection behavior and made an in-depth comparison and analyses of the effects of internal factors and external factors on environmental protection behavior. First of all, the findings reveal the internal mechanism of urban residents’ environmental protection behavior to a certain extent. These findings enrich the relevant existing research results of environmental psychology. In addition, this study expanded the research thinking of previous studies, which only focus on one (or one type of) influencing factor, and thus, this study provides reference for subsequent studies. Environmental protection activities require the participation of the public [16]. Personal and social norms have been shown to have an impact on environmental protection behaviors [17,22,23]. Combining these two factors to discuss their different effects is one of the highlights of this study.

Second, this study conducts a comparative analysis of influencing factors and reveals the different effects of internal and external influences. The comparison of voluntary or forced behavior allows us to find the greater influencing factors of residents’ environmental protection behaviors. These findings enrich previous studies comparing different norms [36].

Third, based on the current level of economic and social development in China and the limited income level of urban residents, cost awareness is included as a moderator in environmental protection behavior influencing factor models. The results further establish the boundary conditions that influence environmental protection behavior, and more comprehensively reveal the complex relationship mechanism between influencing variables. These findings will help deepen the understanding of the relationships between various variables that affect environmental protection behavior. Existing research has confirmed that cost factors not only affect environmental protection behaviors [39,40], but also moderate the impact of factors such as willingness on those behaviors [44]. Studying the effects of cost factors is therefore of great significance, especially in developing countries.

### 7.2. Practical Inspiration

First, the role of educating people about environmental protection should be strengthened, in order to foster a sense of environmental responsibility, particularly in young people. Environmental protection willingness is a key factor that affects residents’ environmental protection behavior. In order to promote environmental protection willingness and generate more environmental protection behavior, we should start with personal norms. In other words, it is necessary to strengthen urban residents’ sense of environmental responsibility, guide urban residents on the path to establish environmental protection values, and allow people to sincerely and deeply realize and understand the importance of environmental protection behaviors. Then, they will consciously and voluntarily practice environmental protection in daily life. Cultivation of environmental responsibility and the establishment of environmental protection values can’t be achieved overnight. We need to educate people from childhood. To this end, we should increase environmental protection courses in elementary and secondary schools, continuously enhance the environmental responsibility of young people, gradually establish young people’s environmental protection values, and vigorously cultivate young people’s environmental behaviors.

Second, environmental protection activities should continually be publicized and promoted throughout the whole of society. Appropriate pressure should be maintained in the form of public opinion with regard to environmental protection. The government should improve the current monitoring mechanisms; environmental protection requirements should not be relaxed. The government should also continue to encourage environmental protection behaviors and actively guide residents to participate in environmental protection. At the same time, policy makers should actively explore the punishment mechanism for any non-environmental behaviors of urban residents, and effective constraints on any urban residents’ harmful environmental protection activities should be formed. For example, in recent years, Australia, Canada, and other countries have implemented varying degrees of restrictions on the use and disposal of plastic. On 1 July 2019, Shanghai, China officially promulgated and implemented the “Shanghai Municipality’s Regulations on the Management of Domestic Waste” to ensure the comprehensive implementation and supervision of waste classification from the legislative level. This measure is conducive to improving the garbage sorting behavior of Shanghai residents.

Thirdly, efforts should be made to reduce environmental protection costs in residents’ daily lives. The cost consciousness factor, as an important moderating variable, has a role that cannot be ignored when residents make environmental choices. The government can promote the occurrence of residents’ positive environmental protection behaviors by reducing the cost of those environmental protection behaviors, or by increasing material rewards. For example, increase subsidies for energy-saving appliances, to reduce the cost for people who want to purchase environmentally friendly products. Appropriately adjust the price range of water and electricity, in order to guide residents’ energy consumption. Publicize and reward citizens who actively engage in and promote environmental protection participation. Finally, set up convenient garbage delivery, classification, and other environmental protection facilities.

### 7.3. Limitations and Future Directions

First, due to the limitation of research resources, this study only selects personal norms and social norms as the measurement indexes of internal and external influencing factors. In the future, more and different measurement indexes can be added to study the path of influence from a more comprehensive and multi-angle perspective.

Second, as the object of this study is urban residents, data collection is difficult. It is especially not easy to collect data from multiple sources or at multiple time points, which affects the internal validity of the study to a certain extent. In the future, diversified data collection and research methods such as conducting longitudinal studies can be adopted. Such methods may lead to more valuable conclusions.

## Figures and Tables

**Table 1:** Reliability and validity analysis results.

Variables		Factor Loading	Cronbach’s α	CR	AVE
Personal norms (PN)	*PN1*	0.821	0.893	0.956	0.815
	*PN2*	0.889			
	*PN3*	0.903			
	*PN4*	0.797			
	*PN5*	0.788			
Social norms (SN)	*SN1*	0.823	0.725	0.907	0.765
	*SN2*	0.855			
	*SN3*	0.726			
Environmental protection willingness (EW)	*EW1*	0.857	0.855	0.945	0.811
	*EW2*	0.850			
	*EW3*	0.872			
	*EW4*	0.767			
Environmental protection behavior (EB)	*EB1*	0.545	0.786	0.889	0.578
	*EB2*	0.510			
	*EB3*	0.642			
	*EB4*	0.789			
	*EB5*	0.800			
	*EB6*	0.715			
Cost consciousness (CC)	*CC1*	0.785	0.681	0.894	0.739
	*CC2*	0.811			
	*CC3*	0.753			

**Table 2:** Pearson correlation coefficient and average variance extraction amount (AVE) square root value of each variable.

Variables	Mean	S.D.	1	2	3	4	5
1.PN	21.399	3.138	(0.903)				
2.SN	11.975	2.122	0.483 **	(0.875)			
3.EW	16.819	2.614	0.566 **	0.514 **	(0.901)		
4.EB	10.679	2.271	0.185 **	0.152 **	0.242 **	(0.760)	
5.CC	11.625	2.237	0.297 **	0.393 **	0.344 **	0.021	(0.860)

Note: ** *p* < 0.01 level. The diagonal is the AVE square root value of the variable, and the lower half of the matrix is the Pearson correlation coefficient.

**Table 3:** Comparison of measurement models.

Model	χ^2^/df	CFI	TLI	RMSEA
Baseline model	4.148	0.914	0.889	0.066
M1	5.901	0.864	0.828	0.082
M2	8.419	0.794	0.740	0.101
M3	5.952	0.862	0.826	0.082
M4	9.387	0.763	0.706	0.107
M5	7.307	0.822	0.779	0.093
M6	11.235	0.711	0.641	0.118
M7	10.475	0.729	0.667	0.114
M8	14.432	0.616	0.529	0.136
M9	12.589	0.669	0.593	0.126

**Table 4:** Regression results of factors affecting environmental protection behavior.

		Dependent Variable: EB			
		M1	M2	M3	M4
Control variable	Gender	−0.006	−0.015	−0.014	−0.010
	Age	0.041	0.027	0.030	0.029
	Occupation	0.071	0.046	0.060	0.051
	Monthly income	0.021	0.008	0.014	0.006
Independent variable	PN		0.179 **		
	SN			0.147 **	
	EW				0.237 **
R^2^		0.006	0.037	0.027	0.062
F-value		1.076	5.566 **	4.069 **	9.522 **
VIF_max_		1.065	1.085	1.071	1.073

Note: ** *p* < 0.01 level. The ordinary least square method is used to calculate the regression equation.

**Table 5:** Results of mediation effect test.

Path	Indirect Effect	S.E.	95% Confidence Interval	
			LL 95% CI	UL 95% CI
PN→EW→EB	0.343 **	0.079	0.187	0.499
SN→EW→EB	0.467 **	0.083	0.306	0.629

Note: ** *p* < 0.01 level. LL 95% CI = lower 95% level confidence interval; UL 95% CI = upper 95% level confidence interval.

**Table 6:** Comparative study results of impact effects.

Parameter	Coefficient	S.E.
r1-r2	1.308 **	0.272
r4a-r4b	1.568 **	0.290
r4a × r3-r4b × r3	1.450 **	0.268

Note: ** *p* < 0.01 level.

**Table 7:** Regression results of the moderating effect of cost consciousness.

		Dependent Variable: EB						
		M1	M2	M3	M4	M5	M6	M7
**Control variable**	Gender	−0.006	−0.014	−0.020	−0.013	−0.013	−0.008	−0.011
	Age	0.041	0.026	0.030	0.029	0.030	0.027	0.030
	Occupation	0.071	0.043	0.045	0.056	0.056	0.044	0.043
	Monthly income	0.021	0.007	0.007	0.013	0.012	0.003	0.000
**Independent variable**	PN		0.186 **	0.212 **				
	SN				0.162 **	0.168 **		
	EW						0.260 **	0.277 **
	CC		−0.026	−0.038	−0.038	−0.042	−0.065 *	−0.075 *
	PN × CC			0.095 *				
	SN × CC					0.024		
	EW × CC							0.067 *
	R^2^	0.006	0.038	0.046	0.028	0.029	0.065	0.069
	F-value	1.076	4.713 **	4.971 **	3.538 **	3.088 **	8.423 **	7.708 **
	VIF_max_	1.065	1.122	1.202	1.207	1.261	1.156	1.223

Note: * *p* < 0.05 level, ** *p* < 0.01 level. The ordinary least square method is used to calculate the regression equation.
